# The reasonable therapeutic modality for biliary duct-to-duct anastomotic stricture after liver transplantation: ERCP or PTC?

**DOI:** 10.3389/fonc.2022.1035722

**Published:** 2022-09-29

**Authors:** Hu Bowen, Guo Wenzhi, Wen Peihao, Shi Jihua, Zhang Shuijun

**Affiliations:** ^1^ Department of Hepatobiliary and Pancreatic Surgery, First Affiliated Hospital of Zhengzhou University, Zhengzhou, China; ^2^ Henan Key Laboratory of Digestive Organ Transplantation, Zhengzhou, China; ^3^ Henan Research Centre for Organ Transplantation, Zhengzhou, China

**Keywords:** biliary complications, anastomotic biliary stricture, ercp, ptc, liver transplantation

## Abstract

**Objective:**

To compare the initial success rate, feasibility, and effectiveness of endoscopic retrograde cholangiopancreatography (ERCP) versus percutaneous transhepatic cholangiography (PTC) for anastomotic biliary stricture after liver transplantation (LT).

**Methods:**

We retrospectively analyzed the data collected during January 2015 to December 2021 from liver transplantation recipients who developed anastomotic biliary stricture after liver transplantation and treated by ERCP and/or PTC. The success rate, complications and patients’ survival rate of ERCP and PTC procedures was evaluated.

**Results:**

Forty-eight patients who underwent LT and were confirmed to have the anastomotic biliary stricture were enrolled. Overall, 48/48 patients underwent single or multiple ERCP procedures as the first line therapy; 121 therapeutic ERCPs (3.36 ± 2.53 ERCPs per patient) were performed in 36/48 patients successfully. All the 12 patients who failed ERCP tend to have special bile duct conditions such as overlong, angle shaped, and/or extremely narrowed bile duct and underwent PTC as an alternative treatment. The initial success rate of ERCP was 75% (36/48) while the success rate of ERCP for the 12 patients with special bile duct was 0% (0/12). PTC was an effective second-line treatment for those 12 patients who failed ERCP, and 58.33% (7 of 12 cases) were treated successfully. The average procedure time in PTC group was significantly lower than ERCP group (*t*=2.292, *P*=0.027). The feasibility of ERCP was associated with the anatomical shape of bile duct and the severity of the stricture site. Finally, the cumulative survival rate was 100% (12/12) in PTC group compared to 86.11% (31/36) in ERCP group (*χ^2 =^* 0.670, *P*=0.413).

**Conclusion:**

ERCP is the gold standard method for the diagnosis and effective intervention for the management of biliary complications after LT. However, its use in certain types of biliary complications (e.g., patients with severe anastomotic biliary stricture and those with overlong and angle shaped bile ducts) is not promising and associated with significant risk of complications. PTC and other interventions should be studied along with ERCP for patients for whom ERCP may not work. The feasibility and efficacy of primary management can be predicted by the noninvasive imaging examinations like Magnetic Resonance Cholangiopancreatography (MRCP) before the procedure, which may help with the choice of the most reasonable therapeutic modality and avoiding unnecessary financial burden and complications.

## Introduction

Hepatocellular carcinoma (HCC) represents an important cause of morbidity and mortality. It is the sixth most common cancer and the fourth leading cause of cancer related death worldwide (1). HCC is increasingly act as a primary indication for liver transplantation. In general, liver transplantation is considered as the best treatment option for early-stage HCC, since it simultaneously treats the tumor and the underlying liver disease (the main risk factor for the development of new tumors). Thus, the number of patients transplanted for HCC are increasing, with a rate of 15-50% in all the liver transplantations performed in the world (2, 3). Although liver transplantation remains an outstanding therapy for HCC, biliary tract complications still remain a common problem following liver transplantation, which has been known as the “Achilles heel” of liver transplantation despite improved surgical technique and experience (4, 5).

Biliary complications after liver transplantation mainly include anastomotic stricture, non-anastomotic stricture, and bile leakage, although other complications such as bile duct stones, sphincter of oddi dysfunction, and progression of primary biliary disease can occur (6–8). These complications are commonly managed by endoscopic retrograde cholangiopancreatography (ERCP), which is considered as the gold standard and first line choice for most kinds of biliary complications after liver transplantation (9–12).

Although ERCP is considered safe and effective, the success rates of ERCP treatment is unsatisfactory, which ranges from 60% to 70% for anastomotic strictures and from 25% to 33% for non-anastomotic strictures (13, 14). For the reason that non-anastomotic biliary stricture has complicated etiology therefore it is quite difficult to be illuminated and managed, we just focused on anastomotic stricture in this study. In addition, the need for multiple ERCPs and post-ERCP complications is relatively high after liver transplantation. The patients for whom ERCP failed should be managed by other alternate techniques. PTC or bilioenteric anastomosis surgery are considered as the second-line therapy for these patients (15–17). However, the success rate, feasibility and effectiveness of PTC has not been well documented. Therefore, the aim of this study is to compare ERCP and PTC therapies in patients with anastomotic stricture.

## Materials and methods

### Patients and data collection

We retrospectively reviewed the study population consisted of 1264 consecutive patients who had undergone liver transplantation. After excluding those who received living donor liver, splitting donor liver, pediatric donor liver, re-transplantation, combined organ transplantation, and T-tube placement, we identified 1171 adult patients who received whole liver Donation after Citizen Death (DCD) at the First Affiliated Hospital of Zhengzhou University. Finally, we included 48 patients who developed anastomotic biliary stricture and underwent ERCP as a primary therapy for the management. No organ was obtained or used from executed prisoners in this study. All recipients received a duct-to-duct anastomosis of the bile duct without T-tube.

**Figure 1:**
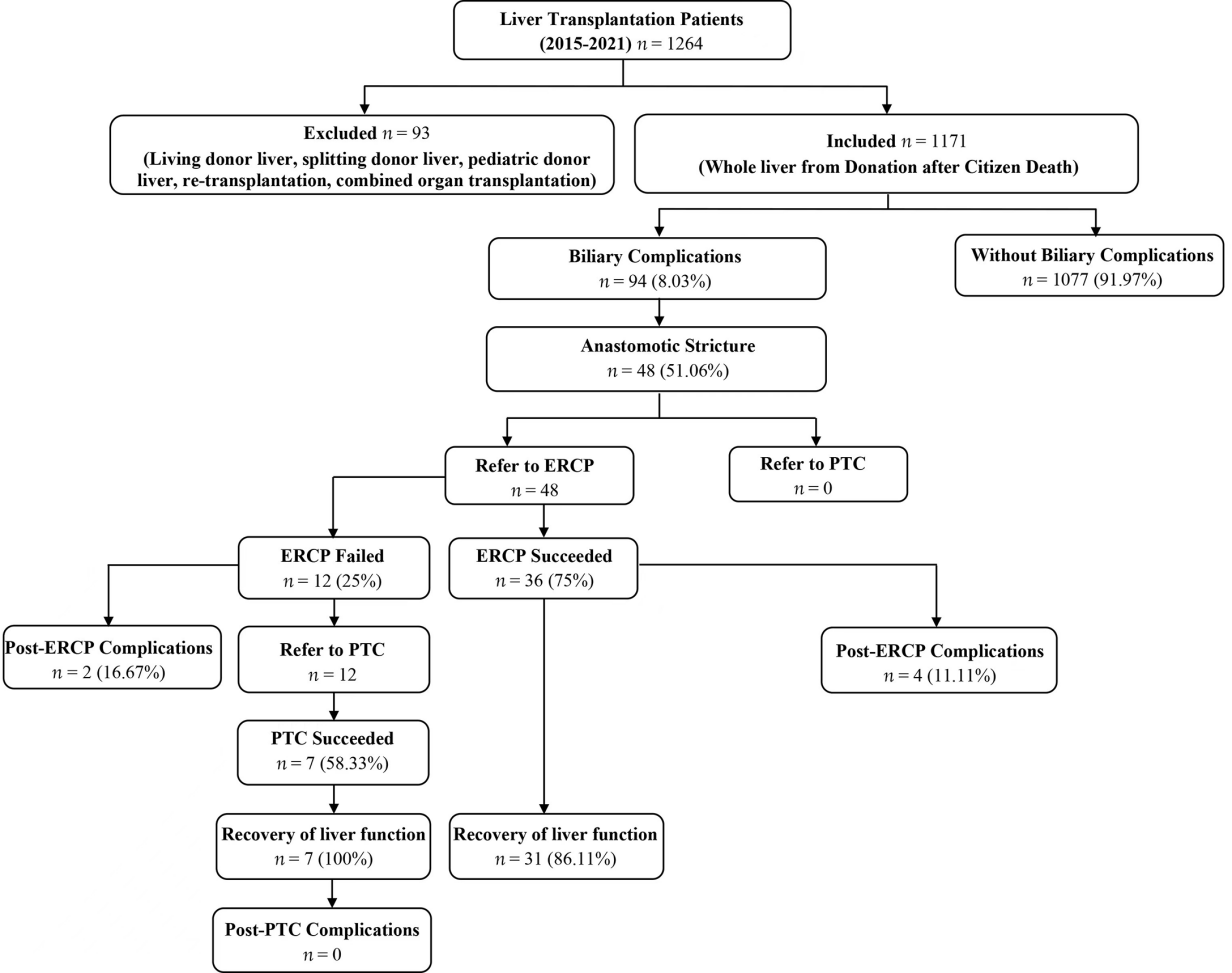
Schematic of study design and procedural data of liver transplantation patients.

**Figure 2:**
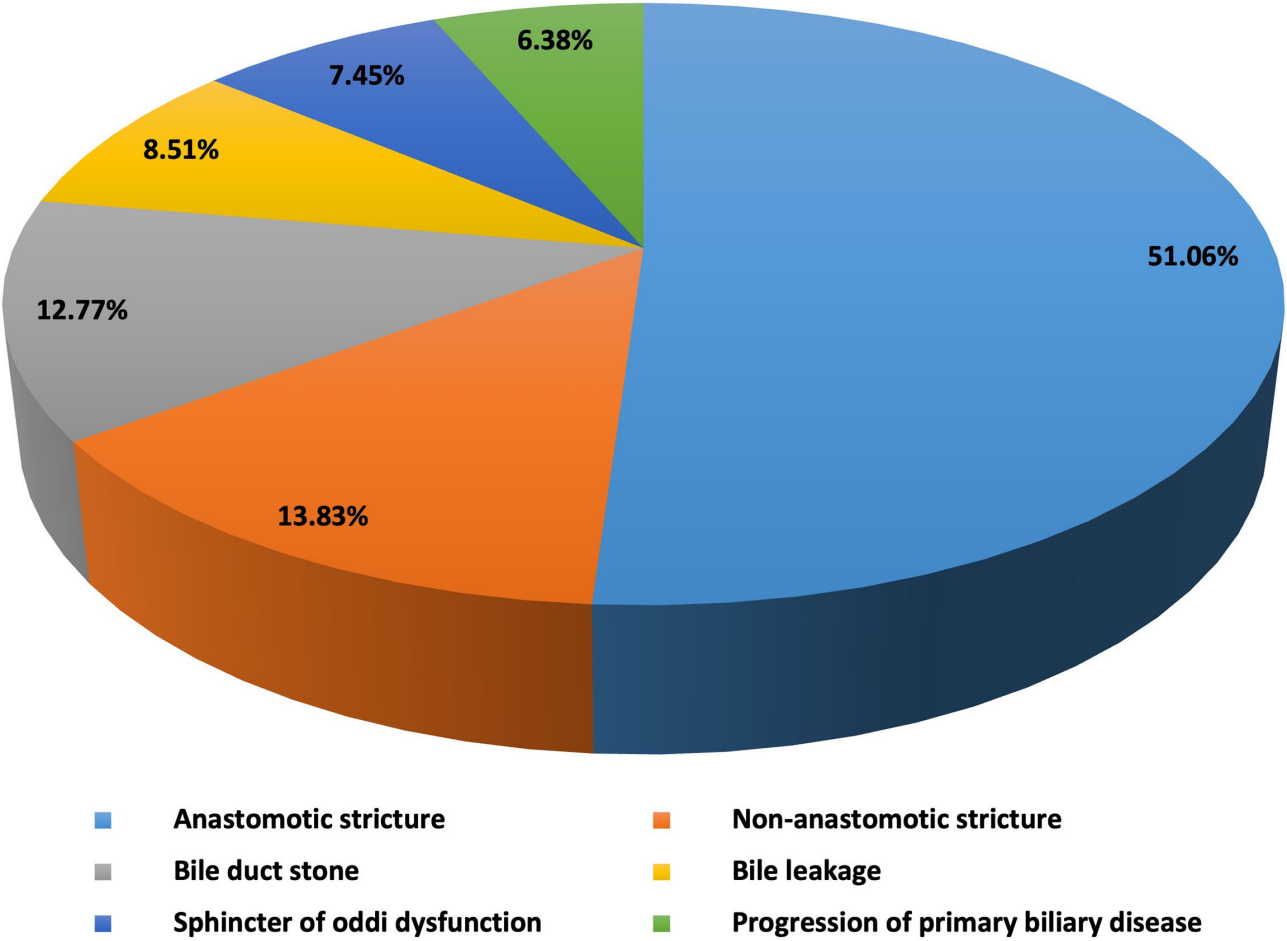
Baseline characteristics (etiology) of included patients.

The study protocol was approved by the Institutional Review Board of the first affiliated hospital of Zhengzhou university and all methods were performed in accordance to relevant guidelines and regulations. Informed written consent was obtained from each patient before performing all procedures. We recorded the relevant demographic details (age, gender), etiology, biliary complications after liver transplantation, number of ERCPs and PTCs per patient, post-ERCP and PTC complications, and complementary or alternative treatments to ERCP (e.g., PTC and/or surgery) (**Figure 1**). Liver function of all the included patients were recorded and analyzed before and after the procedures. The baseline characteristics of included patients are shown in **Table 1** and **Figure 2**.

**Table 1:** Baseline characteristics of 1171 liver transplantation patients.

	Biliary complication	Without Biliary complication	*Z/χ^2^*	*P* value
	n = 94	n = 1077		
**Age (years), Median (P25, P75)**	50 (25, 57)	49 (43, 56)	0.689	0.491
**Gender**				
Male (%)	68 (72.3)	914 (84.9)	10.020	0.002
Female (%)	26 (27.7)	163 (15.1)		
**Indications for liver transplantation (%), (n=1171)**				
Hepatocellular carcinoma (HCC)	36.2%		424	
Hepatitis B cirrhosis	34.2%		400	
Alcoholic cirrhosis	7.9%		93	
Other disease	7.2%		84	
Autoimmune (AIH, PBC, PSC)	5.9%		69	
Acute liver failure	3.7%		43	
Hepatitis C cirrhosis	1.7%		20	
Wilson disease	1.1%		13	
Hilar cholangiocarcinoma	0.9%		10	
Hepatitis B and C cirrhosis	0.7%		8	
Cholangiocellular carcinoma	0.5%		6	
Amyloid polyneuropathy	0.1%		1	
**Biliary complications (%), (n=94)**				
Anastomotic stricture	51.06%		48	
Non-anastomotic stricture	13.83%		13	
Bile duct stone	12.77%		12	
Bile leakage	8.51%		8	
Sphincter of oddi dysfunction	7.45%		7	
Progression of primary biliary disease	6.38%		6	

AIH, autoimmune hepatitis; PBC, primary biliary cholangitis; PSC, primary sclerosing cholangitis. Other disease, Budd-Chiari, Polycystic liver, Cryptogenic cirrhosis.

### Interventions

All ERCPs were performed by experienced endoscopists with a volume of 300 to 500 ERCP procedures annually. Based on the radiological findings and the clinical characteristics of the patients, the type of ERCP treatment (ENBD or ERBD) and the diameter, size, number, and frequency of stent replacement were determined by the endoscopist.

An anastomotic stricture was defined as a dominant, short narrowing at the site of anastomosis and the contrast medium pass through narrowly or cannot pass through the stricture site on MRCP or ERCP imaging. ERCP procedure was considered to be accomplished successfully when the guide wire pass through the stricture site and a biliary stent was placed across the stricture site successfully during the ERCP procedure. ERCP was failed when the ERCP procedure was not completed according to the original plan (i.e., the guide wire cannot pass through the stricture site successfully). Patients who failed ERCP were managed by alternative or complementary treatment options such as PTC, surgery, or both.

### Statistical analysis

The statistical analysis was performed using IBM SPSS Statistics 24.0. Descriptive statistics were employed to report findings, continuous variables were reported as means with standard deviation (SD) or median, and interquartile range (IQR). Categorical variables were reported as a percentage (%). The comparison of the differences in major characteristics between biliary complication and non-biliary complication groups was examined by t-tests, Wilcoxon Mann–Whitney test, or chi-square test as appropriate. A Kaplan-Meier survival analysis was also conducted. The statistical significance level was 0.05 for a two-tailed test.

## Results

**Figure 3:**
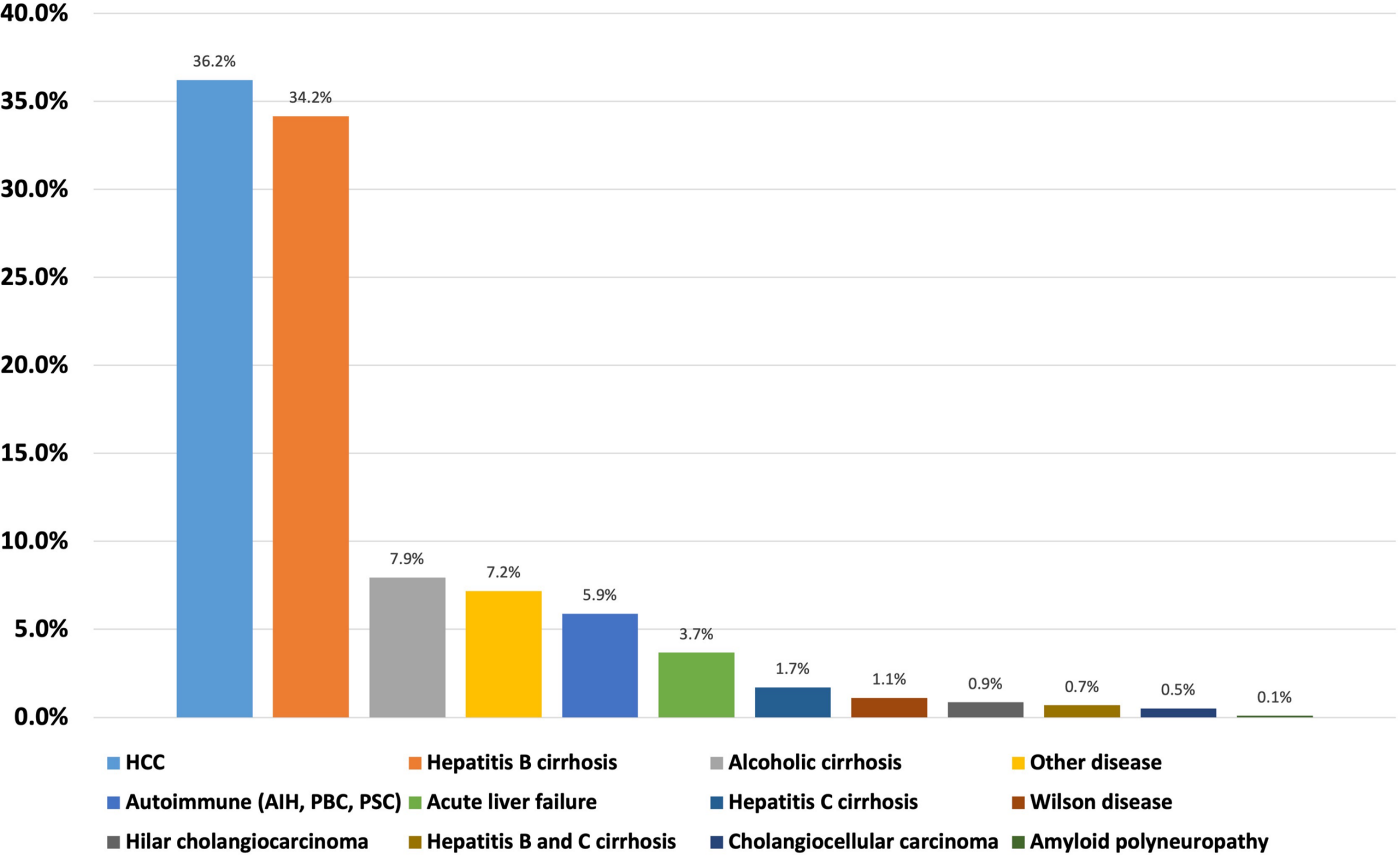
Biliary complications ratios after liver transplantation.

We identified 1171 cases (age, mean ± SD: 49.40 ± 10.26 years; range 18-75), 83.86% men. Ninety-four of 1171 (8%) patients developed biliary complications during a follow-up of 81.6 months (mean ± SD: 38.20 ± 25.31 months). The main reported biliary complications were anastomotic strictures in 51.06% (48/94) patients (**Figure 3**). Therapeutic ERCP was performed in 48/48 patients as a first line therapy. Overall, 121 therapeutic ERCPs (mean 3.36 ± 2.53 ERCPs per patient) were performed in 36/48 patients with biliary anastomotic stricture who gained success from ERCP. The initial success rate of ERCP was 75% (36/48). Twelve patients who failed ERCP were found to have special bile duct conditions such as overlong, angle shaped, and/or extremely narrowed bile duct on MRCP and ERCP examination, and later were allocated to PTC procedure as a second line therapy. The success rate of ERCP in patients with special bile duct was 0% (0/12), while the initial success rate of PTC in patients with special bile duct was 58.33% (7/12). Totally only 8 therapeutic PTCs (mean 1.14 ± 0.38 PTCs per patient) were performed in 7/12 patients who gained success from PTC, which was significantly lower than ERCP group (*t*=2.292, *P*=0.027). Five of 12 (41.67%) patients with special bile ducts who failed PTC received biliary enteric anastomosis as their final therapy.

Six patients suffered post-ERCP complications consist of cholangitis in 3, pancreatitis in 2 and bile leakage in 1 patient. Unfortunately, 5 of 36 patients who gained ERCP successfully died before the endpoint date of our study, of which 4 (80%) patients suffered from post-ERCP complications. We believed that complications caused by ERCP maybe one of the risk factors leading to mortality of these patients. However, there could be other risk factors such as liver function cannot be alleviated by ERCP procedure and therefore led to multi-organ functional disturbance ultimately. Importantly, the 12 patients with special bile duct conditions of which 7 received PTC successfully and 5 received biliary enteric anastomosis did not have any complications nor morbidity until our study endpoint.

The liver function of patients who underwent ERCP and PTCD were examined and analyzed before and after the procedure. In PTC group, all the patients 7/7 (100%) who underwent successful PTC gained recovery of liver function. On the other hand, in ERCP group, only 31/36 (86.11%) patients who received successful ERCP gained the recovery of liver function and the remaining 5/36 patients died finally. Those 5/36 patients whose liver function failed to recover after successful ERCP procedure may cause by the occlusion of the single plastic stent etc. Therefore, it is suggested that ERCP may not be the best choice for every patient suffered from biliary anastomotic stricture, especially for those with special bile duct conditions that failed ERCP. PTC is more efficient than ERCP for those patients with overlong, angle shaped or severe stricture bile duct that failed ERCP procedure (58.33% vs 0%).

**Figure 4:**
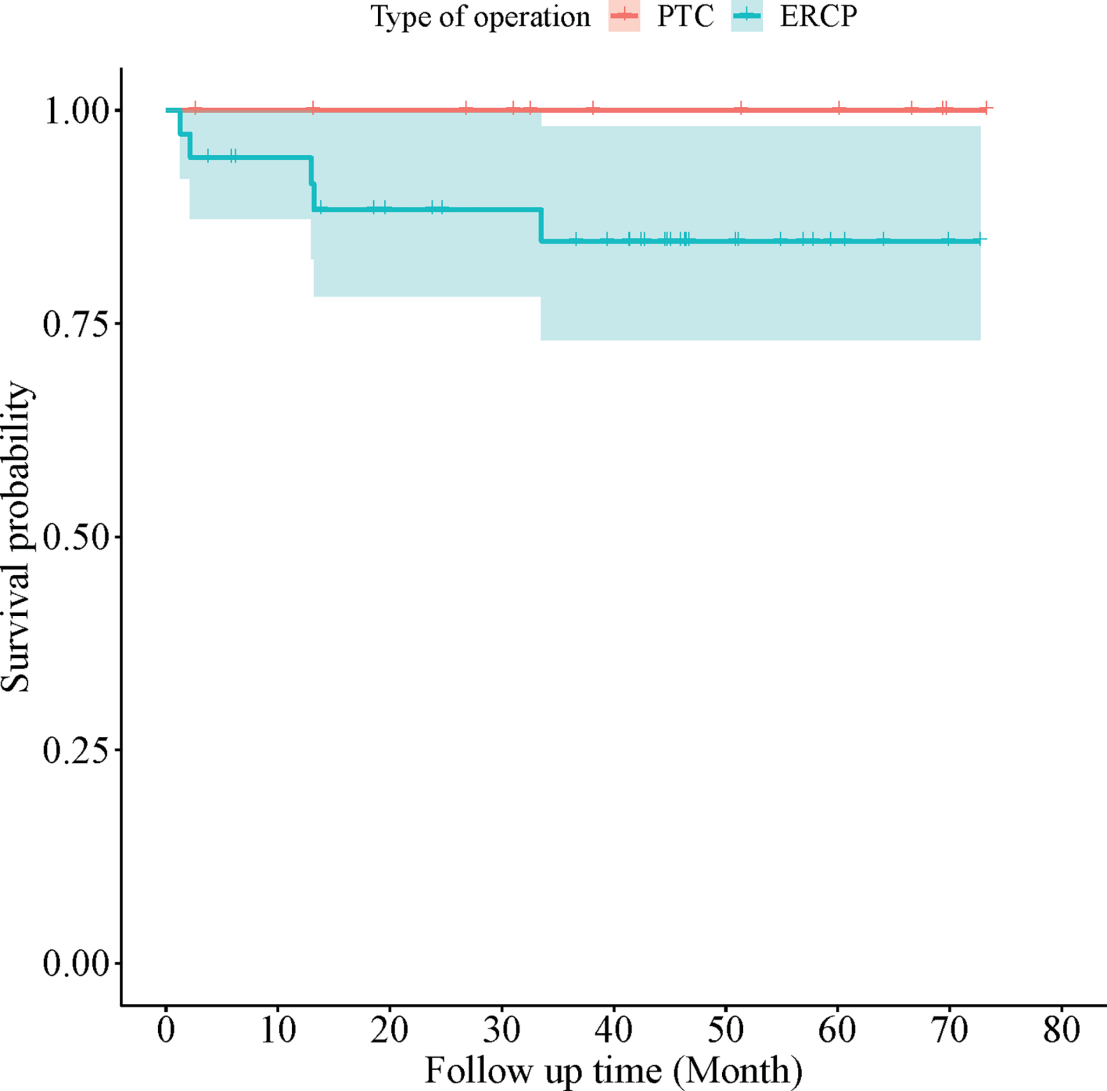
Kaplan-Meier survival analysis in 36 ERCP patients and 12 PTC patients.

The cumulative survival rate of the 36 patients who underwent successful ERCP was 86.11% (mean: 63.71, 95% CI: 56.35-71.08 months) compared to 100% (mean: 73.30, 95% CI: 73.30-73.30 months) of those underwent PTC (*χ^2 =^* 0.670, *P*=0.413) (**Figure 4**). Patients underwent PTC had better overall survival compared to the patients who underwent ERCP successfully.

## Discussion

Biliary tract complications are commonly recognized clinically and considered as an important cause of morbidity and mortality after liver transplantation with an estimated incidence of 5% to 35% worldwide (3–7). Benign biliary strictures after transplantation can be classified into anastomotic and non-anastomotic strictures, which play an important role in graft and patient survival. For the reason that non-anastomotic biliary stricture has complicated etiology and is quite difficult to be illuminated and well managed, we just focused on anastomotic stricture in this study. Currently, biliary stricture is commonly managed by the gold standard and first line therapy ERCP for its efficient and minimally invasive character. However, other procedures including PTC intervention or surgery can also be considered if the condition cannot be managed by ERCP successfully (13–16). ERCP is a highly effective therapy for biliary complications after liver transplantation, but in some cases, the initial therapy may fail because of the inability of guide wire to pass through the stricture. In this study, we mainly evaluated the success rate, complications rate, patient’s survival rate, and efficacy of ERCP and PTC procedures for anastomotic stricture.

The overall incidence of biliary strictures ranges from 10%-37% after liver transplantation, and anastomotic stricture comprises the majority of biliary strictures (8, 10, 16, 17). In our study, we found that 64.89% of the biliary complications was biliary stricture; of which 78.69% were anastomotic stricture, which was in accordance with previous studies. Several previous studies confirmed that ERCP and biliary stenting is a successful treatment in the majority of patients, which is considered to be more minimally invasive and convenient compared to both PTC and surgical treatment. Nevertheless, there are some procedure failures and also some contraindications, such as a minimal time between the liver transplantation surgery and ERCP procedure (8–10, 18, 19).

**Figure 5:**
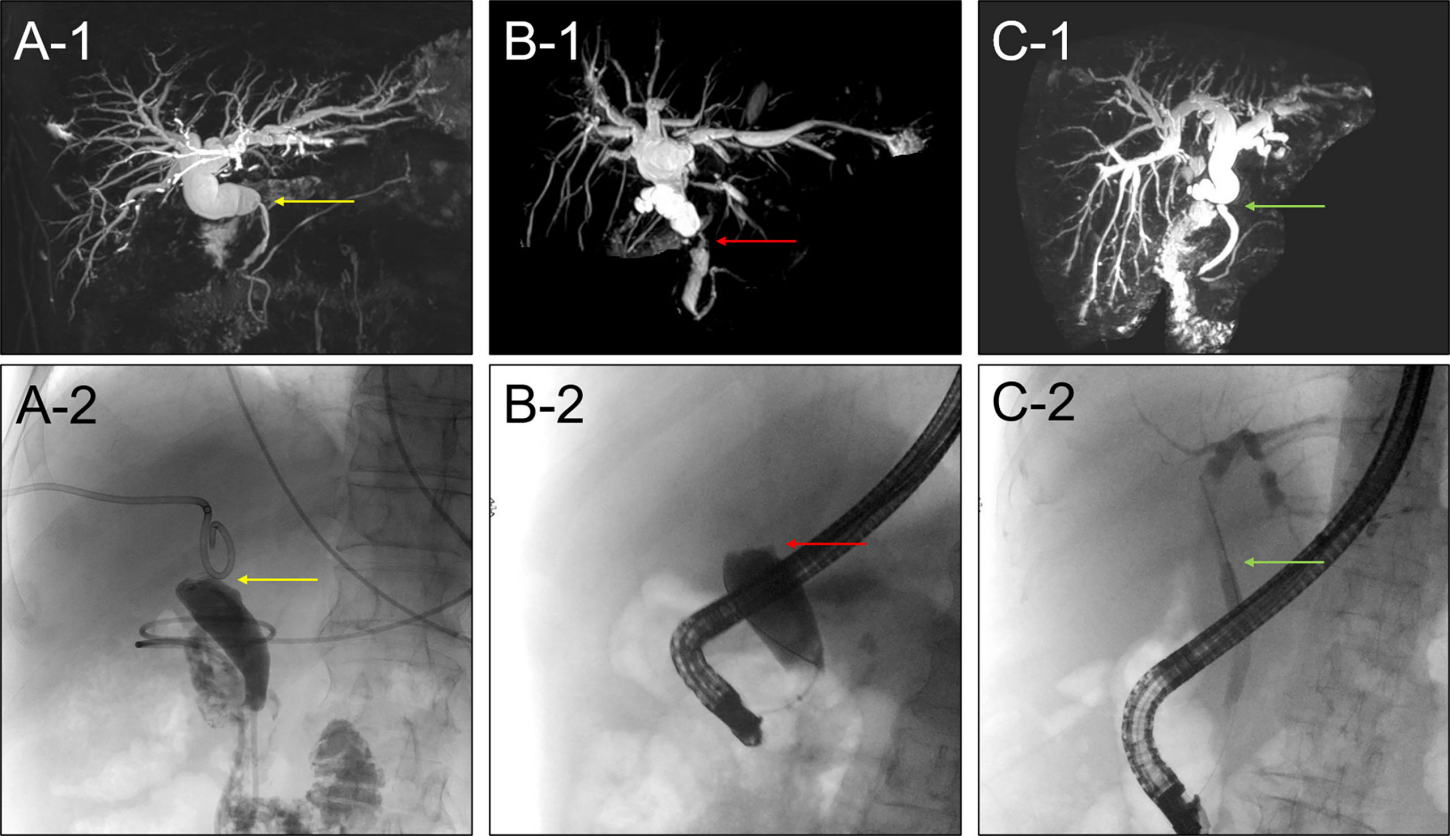
**(A1)** MRCP image showed the anastomotic biliary stricture with overlong and angle shaped bile duct. **(A2)** ERCP failed in patients with overlong and angle shaped bile duct. **(B1)** MRCP image showed the severe anastomotic biliary stricture. **(B2)** ERCP failed in patients with severe anastomotic biliary stricture. **(C1)** MRCP image showed a simple and moderate anastomotic biliary stricture. **(C2)** ERCP succeed in patients with simple and moderate anastomotic biliary stricture.

We have observed that ERCP failed in patients with overlong, angle shaped bile duct and severe anastomotic biliary stricture, which didn’t not allow the contrast medium and guide wire to pass through during ERCP (**Figures 5A1, B1**). However, PTC got success under these circumstances. On the other hand, ERCP is appropriate in patients with simple and moderate anastomotic biliary stricture, which allows the guide wire to pass through (**Figures 5C1, 5B2**). Most patients with anastomotic stricture require multiple endoscopic sessions at a frequency of every 2-3 months and the placement of single or multiple stents of 7-11.5 Fr for at least 12-24 months to prevent stent occlusion and other post-ERCP complications. In this study, we used a single plastic stent (TTSO-8.5-8, Cook, USA), however, some recent studies indicate that multiple plastic stents or metallic stents are comparatively more efficient, suggesting that the type of stent might be one of the factors affecting the efficiency of ERCP and the need for multiple ERCPs (20–22). Another endoscopic approach, defined by the placement of a fully covered self-expandable metal stent across the stenosis, has been reported to be effective; however, the high migration rate remains to be a major concern (23, 24). Further studies are needed before a definitive conclusion is drawn on which type of stent placement is the best choice for anastomotic biliary stricture after liver transplantation. However, we believed that the type of stent could be directly correlated with the success rate and post-ERCP complications. We have realized that stent placement is suitable for anastomotic stricture, while endoscopic nasal biliary drainage (ENBD) could be preferred for leakage patients. Nevertheless, further investigations are deemed necessary to evaluate the suitability of stent placement or ENBD for leakage patients.

PTC is usually reserved for severe anastomotic biliary stricture or overlong and angle shaped bile duct which cannot be traversed by ERCP and for patients who have undergone Roux-en-Y reconstruction after liver transplantation (**Figures 5A2, C2**). Although usually successful, PTC therapy is regarded as a second-line alternative therapy because of its invasive character, which may cause the discomfort and inconvenience for the patients. Gwon et al. (25) recently developed a technique using the dual catheter placement technique, namely 2 drainage catheters inserted *via* a single percutaneous tract. They achieved clinical success in 98.7% of 79 patients with anastomotic stricture. Our study showed that PTC was successful in 58.33% (7/12) cases where ERCP was totally failed. This is considerable success rate of 58.33% compared to 0% of ERCP for the 12 patients with special bile duct condition who failed the ERCP. So we believed that for the patients with severe stricture or angle shaped bile duct, PTC could be the primarily alternative therapy to ERCP. PTC treatment instead of ERCP will not only avoid need for multiple ERCPs and post ERCP complications but also have potential to reduce the patients’ visit to the hospital and extra financial burden. The feasibility of primary management can be predicted by the MRCP imaging findings, which may help with the choice of the therapeutic modality at the first place itself. If MRCP findings showed the severe stricture or the bile duct is too long, or there is an angulation at the stricture site, we recommend PTC as the first line therapy. However, this study has several limitations including retrospective nature of the study, lack of control group, small sample size, and short follow up time. We believe that it is important to share initial results with fellow colleague so they would know what to expect and what more can be done to improve the technique.

In conclusion, ERCP is the gold standard for the diagnosis and effective intervention for the management of most kinds of biliary complications after liver transplantation. ERCP should be preferred, whenever feasible with the aim to avoid surgical intervention and resolve the patients’ problems. However, its use in some cases was not promising and the need for multiple ERCPs is relatively high; therefore, it should not be the best option for certain kinds of biliary complications (e.g., patients with severe anastomotic biliary stricture and/or overlong and angle shaped bile duct). Beside this, a high proportion of these patients will need PTC/surgery as their final therapy. PTC and other interventions should be studied along with ERCP for patients for whom ERCP may not work. The feasibility of primary management can be predicted by the cholangiographic findings like MRCP, which may help with the choice of the therapeutic modality and avoid unnecessary complications after ERCP and extra financial cost. Further prospective, multicenter studies are needed to confirm these results.

## Data availability statement

The raw data supporting the conclusions of this article will be made available by the authors, without undue reservation.

## Ethics statement 

The studies involving human participants were reviewed and approved by The First Affiliated Hospital of Zhengzhou University. The patients/participants provided their written informed consent to participate in this study.

## Author contributions

Study concept and design: HB, ZS. Analysis and interpretation of data: HB, GW, WP, SJ. Acquisition of data: WP, SJ. Administrative, technical or material support: HB, GW, WP, SJ. Critical revision of the manuscript: ZS. All authors contributed to the article and approved the submitted version.

## Funding

1. Hepatobiliary foundation of Henan Charity General Federation (No: GDXZ2019006). 2. Youth Project of the National Natural Science Foundation of China (82103282).

## Conflict of interest

The authors declare that the research was conducted in the absence of any commercial or financial relationships that could be construed as a potential conflict of interest.

## Publisher’s note

All claims expressed in this article are solely those of the authors and do not necessarily represent those of their affiliated organizations, or those of the publisher, the editors and the reviewers. Any product that may be evaluated in this article, or claim that may be made by its manufacturer, is not guaranteed or endorsed by the publisher.
